# Initial supplementary oxygen concentration for moderate-late preterm infants receiving respiratory support in the delivery room: study protocol for the multicenter, cluster-randomized, crossover AIROPLANE trial

**DOI:** 10.1186/s13063-025-09238-2

**Published:** 2025-12-24

**Authors:** Stacey Peart, Brett J. Manley, Jeanie L. Y. Cheong, Ju Lee Oei, Anneke Grobler, Li Huang, Peter G. Davis, Louise S. Owen

**Affiliations:** 1 Newborn Services and Newborn Research, Royal Women’s Hospital, Melbourne, Australia; 2 Department of Obstetrics, Gynaecology and Newborn Health, The University of Melbourne, Melbourne, Australia; 3 Clinical Sciences, Murdoch Children’s Research Institute, Melbourne, Australia; 4 Department of Paediatrics, Mercy Hospital for Women, Melbourne, Australia; 5 National Health and Research Medical Council Clinical Trials Centre, The University of Sydney, Sydney, New South Wales Australia; 6 Department of Newborn Care, The Royal Hospital for Women, Randwick, New South Wales Australia; 7 Discipline of Paediatrics and Child Health, School of Clinical Medicine, University of New South Wales, Sydney, New South Wales Australia; 8 Department of Paediatrics, The University of Melbourne, Melbourne, Australia; 9 Clinical Epidemiology and Biostatistics Unit, Murdoch Children’s Research Institute, Melbourne, Australia; 10 Melbourne School of Population and Global Health, The University of Melbourne, Melbourne, , Australia

**Keywords:** Infant and preterm, Respiratory distress syndrome, Oxygen, Neonatal intensive care, Delivery room, Birth, Resuscitation, Cluster randomized

## Abstract

**Background:**

Moderate-late preterm infants born at 32–35 completed weeks’ gestation constitute a large proportion of all preterm births (< 37-week gestation), yet they are not well represented in the newborn resuscitation literature. Preterm infants often receive respiratory support in the delivery room, and recommendations exist to guide the use of supplemental oxygen when providing this support. However, there are minimal data regarding the best initial supplementary oxygen concentration for moderate-late preterm infants requiring respiratory support at birth, resulting in practice variation. The aim of this trial is to compare initial supplementary oxygen concentrations of 30% and 21% (air) in preterm infants of 32–35 weeks’ gestation who require respiratory support in the delivery room, with a primary outcome of the need for ongoing respiratory support upon leaving the delivery room.

**Methods:**

This is a prospective, unblinded, multicenter, cluster-randomized, crossover trial in Australian maternity hospitals comparing initial supplementary oxygen concentrations of 30% and 21% (air) in moderate-late preterm infants who require respiratory support at birth. Eligible infants are those born from 32 + 0 to 35 + 6 weeks’ gestation without major cardiorespiratory or craniofacial anomalies, who are receiving active care, and who receive respiratory support in the delivery room within the first three minutes after birth. The primary outcome is the need for ongoing respiratory support upon leaving the delivery room. The trial will recruit a minimum of 1200 infants from at least 20 study sites in Australia using a waiver of consent process.

**Discussion:**

The AIROPLANE trial is a pragmatic study in an underrepresented population using a novel study design. It will be the first randomized study of initial supplementary oxygen concentration during respiratory support in the delivery room to exclusively recruit moderate-late preterm infants. The results will address an important evidence gap and will inform future international guidelines.

**Trial registration:**

Australian New Zealand Clinical Trials Registry ACTRN12621001267842. Registered on 20th September 2021.

## Administrative information

**Table:** 

Title {1}	Initial supplementary oxygen concentration for moderate-late preterm infants receiving respiratory support in the delivery room: study protocol for the multicenter, cluster-randomized crossover AIROPLANE trial
Trial registration {2a and 2b}.	Australian New Zealand Clinical Trials Registry (https://www.anzctr.org.au): ACTRN12621001267842. Registered on 20th September 2021 prior to the first patient being enrolled. Contact for public enquiries: airoplane.trial@mcri.edu.au Contact for scientific enquiries: louise.owen@thewomens.org.au All items from the WHO Trial Registration Data Set can be found within this protocol.
Protocol version {3}	Protocol Version 6.0, 13th February 2024
Funding {4}	•1. Centre of Research Excellence (CRE) in Newborn Medicine, MCRI (National Health and Medical Research Council (NHMRC) no. 1153176) •2. The University of Melbourne, Department of Obstetrics, Gynaecology and Newborn Health, Innovation Grant Scheme •3. The University of Melbourne, Department of Obstetrics, Gynaecology and Newborn Health, Rowden White Trust •4. NHMRC Investigator Grants (Owen no. 2017734 and Manley no. 2016662)
Author details {5a}	S. P., B. J. M., J. L. Y. C., P. G. D., and L. S. O.: Department of Obstetrics, Gynaecology and Newborn Health, The University of Melbourne, Melbourne, Australia; Murdoch Children’s Research Institute, Melbourne, Australia S. P., J. L. Y. C., P. G. D., and L. S. O.: Newborn Services and Newborn Research, The Royal Women’s Hospital, Melbourne, Australia B. M.: Department of Paediatrics, Mercy Hospital for Women, Melbourne, Australia J. O.: National Health and Research Medical Council Clinical Trials Centre, The University of Sydney, New South Wales, Australia; Department of Newborn Care, The Royal Hospital for Women, Randwick, New South Wales, Australia; Discipline of Paediatrics and Child Health, School of Clinical Medicine, University of New South Wales, New South Wales, Australia A. G.: Murdoch Children’s Research Institute, Melbourne, Australia; Department of Paediatrics, University of Melbourne L. H.: Melbourne School of Population and Global Health, The University of Melbourne, Melbourne, Australia
Name and contact information for the trial sponsor {5b}	Melbourne Children’s Trials Centre, Murdoch Children’s Research Institute (MCRI), Melbourne, Australia (mctc@mcri.edu.au)
Role of sponsor {5c}	The trial sponsor is responsible for the initiation and oversight of the trial and carries medicolegal responsibility regarding its conduct. The trial sponsor is also responsible for ensuring that appropriate approvals are obtained prior to the commencement of the trial, that conditions of any approvals are adhered to during the course of the trial, and that the ethical principles of research merit and integrity, justice, beneficence, and respect are applied to the conduct of the trial. The sponsor also ensures that trial compliance is monitored appropriately.

## Introduction

### Background and rationale {6a}

Globally, every year, 15 million babies are born preterm (before 37 weeks’ gestation), equating to approximately 10% of all live births [1]. “Moderate-late preterm” infants, defined as those born from 32 weeks to 36 completed weeks’ gestational age (GA), constitute the majority of all preterm births [2]. However, these infants are poorly represented in the newborn resuscitation literature compared with infants born at term or those born “extremely preterm” before 28 weeks’ gestation, the result of which is a lack of evidence-based resuscitation guidelines for this sub-group of preterm infants.

The initial supplemental oxygen concentration used when providing respiratory support in the delivery room has been the focus of resuscitation research over the past three decades. Studies have demonstrated that initiating respiratory support in term infants using 21% supplemental oxygen (air), compared with 100% oxygen, is associated with reduced mortality [3], and the International Liaison Committee on Resuscitation (ILCOR) suggests commencing with 21% oxygen for infants ≥ 35 weeks’ gestation [4]. A meta-analysis by Welsford et al. demonstrated that almost all “very” preterm infants born before 32 weeks’ gestation who receive delivery room respiratory support starting with air (21% oxygen) go on to receive supplemental oxygen within the first 10 min after birth [5]. This finding is consistent with studies reporting that commencing respiratory support with supplemental oxygen increases spontaneous respiratory effort in animals and humans and may reduce the need for ongoing respiratory support [6, 7]. However, the concentration of supplemental oxygen for commencing resuscitation in preterm infants remains unclear. A Cochrane review comparing the use of low (< 40%) versus high (> 40%) initial oxygen concentration to resuscitate preterm infants at birth found no difference in mortality [8].

However, it is established that resuscitation using high oxygen concentrations causes adverse effects in both term and preterm infants, through the generation of oxygen-free radicals [9–11]. With a lack of evidence for the benefit, and possible evidence of harm, with use of high oxygen concentrations, ILCOR currently suggests the initial use of 21–30% supplemental oxygen when commencing resuscitation of preterm infants born < 35 weeks’ gestation. In contrast, the European Resuscitation Council (ERC) currently recommends respiratory support commencing with 21% oxygen for infants born > 32 weeks’ gestation, 30% supplemental oxygen for infants born < 28 weeks’ gestation, and 21–30% supplemental oxygen for infants between 28 and 32 weeks’ gestation [12]. These differences lead to inconsistent guidelines for infants born at 32–35 weeks’ gestation.

A major reason for the lack of a universal guideline for infants born between 32 and 35 weeks is the paucity of evidence. Welsford et al. [5] identified only 4 small studies addressing this topic; these studies included a total of 296 infants less than 35 weeks’ GA. However, the mean gestational age of all enrolled infants within these four studies ranged from 29 to 32 weeks, reflecting that they mostly enrolled more immature cohorts. To date, no study has specifically addressed the question of initial oxygen concentration in infants born between 32 and 35 weeks’ gestation.

This lack of evidence is reflected in clinical practice, where there is wide variation observed. In a recent international survey conducted by Sotiropoulos et al. [13] in 21 high- and middle-income countries, 42% of neonatologists reported initiating resuscitation of moderate-late preterm infants with 21% oxygen, 35% commenced with 30% oxygen, and the remainder with higher oxygen concentrations (including 3% who used 100% oxygen). In preparation for this trial, we conducted a survey of 25 maternity hospitals in the states of Victoria and New South Wales, Australia. Our survey found similar variation, with 44% of respondents commencing respiratory support with 21% oxygen for infants 32–35 weeks’ gestation and 24% commencing with 30% oxygen, and the final 32% stated there was clinician discretion to use 21–30% oxygen (unpublished).

## Objectives {7}

### Aim

The aim of the AIROPLANE trial is to determine, in infants born between 32 + 0 and 35 + 6 weeks’ gestation, who require respiratory support at birth and whether commencing support in 30% oxygen or 21% oxygen reduces the need for ongoing respiratory support upon leaving the delivery room.

### Hypothesis

In infants born between 32 + 0 and 35 + 6 weeks’ gestation who require respiratory support at birth, commencing with 30% oxygen, compared with 21% oxygen, will reduce the need for ongoing respiratory support.

## Trial design {8}

AIROPLANE is a phase 3, interventional, and preventative trial. It is a prospective, unblinded, multicenter, cluster-randomized crossover, superiority trial recruiting in Australian maternity hospitals.

## Methods: participants, interventions and outcomes

### Study setting {9}

The study will be conducted in a minimum of 20 maternity hospitals in Victoria (VIC) and New South Wales (NSW), Australia, comprising a mixture of tertiary metropolitan, non-tertiary metropolitan, and regional centers.

## Eligibility criteria {10}

Inclusion criteria (all must apply) are as follows:

1. Born between 32 + 0 and 35 + 6 weeks’ gestation
2. Receiving active care
3. Requiring respiratory support in the first three minutes after birth

The only exclusion criterion is the antenatal diagnosis of a major cardiorespiratory or craniofacial anomaly.

### Who will take informed consent? {26a}

Both AIROPLANE trial treatment arms are current standard medical treatments provided in an emergency setting delivered under the Australian Medical Treatment Decision & Planning Act (2016), and as such do not require prior consent for use at any study sites. All but one study site falls under a single Royal Children’s Hospital Human Research Ethics Committee (HREC) that approved waived consent for data collection, transfer, and analysis (Reference 78071). The Mercy Hospital for Women operates under a separate HREC that approved a formal opt-out approach for data analysis (Reference 2021-073). At that site, eligible infants receive the study treatment and undergo initial data collection; then, prior to hospital discharge and further data collection, parents are given the opportunity to opt out of data analysis and have their infant’s data deleted. A public disclosure program will be implemented to inform families of their child’s enrolment in the study. This includes posters displayed in all participating sites, information brochures provided to families, and an open website (https://www.airoplanetrial.org.au) containing an informational video about the trial.

### Additional consent provisions for collection and use of participant data and biological specimens {26b}

Collection of maternal surname and infant identifiers (unique hospital number, date of birth, and time of birth) is included under the approved waiver of consent. This is limited at one site, where maternal surname will not be collected.

For infants born in Victoria, some infants will be prospectively consented and co-enrolled into the Generation Victoria project (GenV.org.au). This separate, epidemiological study will provide extended in-patient and long-term secondary outcome data for co-enrolled AIROPLANE trial participants. The maternal and infant identifiers will enable identification of co-enrolled infants into GenV. GenV consent includes data linkage and data access for approved trials. The approved AIROPLANE consent waiver includes data linkage with the GenV database for co-enrolled infants. The GenV project is a separate study, with a stand-alone protocol and results that will be reported separately. Extended and long-term secondary data for co-enrolled infants will be reported separately following publication of the main trial results.

## Interventions

### Explanation for the choice of comparators {6b}

Currently, 21% oxygen (air) and 30% oxygen are considered standard practice in units providing respiratory support to moderate-late preterm infants at birth, both locally and internationally. There is no evidence to suggest that one may be superior to the other, international guidelines are contradictory, and both international and local clinical equipoise exists.

### Intervention description {11a}

AIROPLANE is a cluster, randomized crossover trial where the unit of randomization is the hospital, rather than the individual infant. Hospitals will be randomly allocated to one of two treatment arms for a set duration: treatment A: 21% oxygen or treatment B: 30% oxygen. Hospitals will then crossover to provide the alternate treatment arm for the same set duration.

**Fig. 1:**
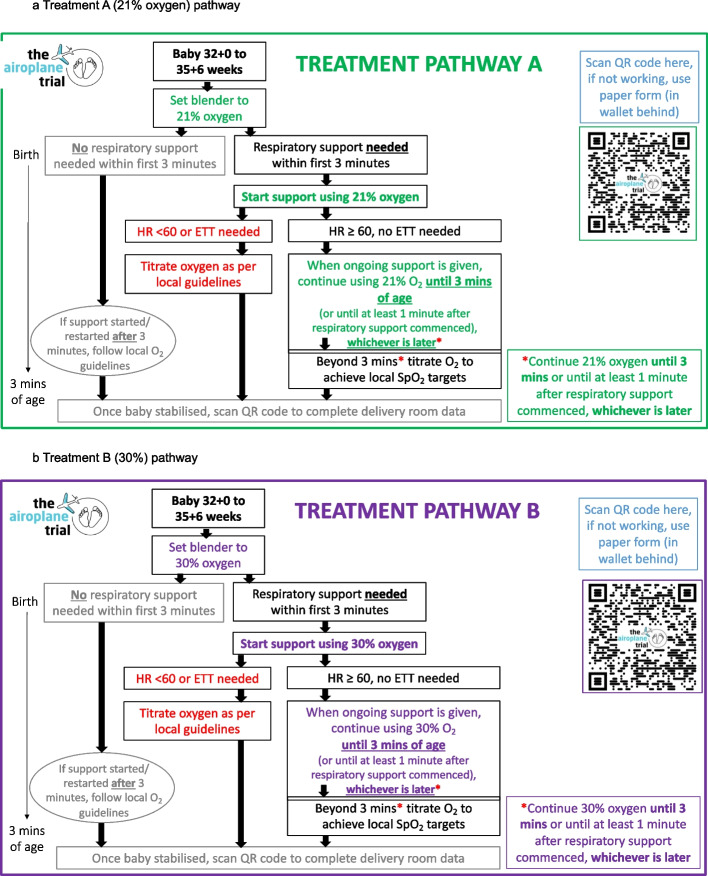
**a** Treatment A (21% oxygen) pathway. **b** Treatment B (30% oxygen) pathway

Following birth, infants who require any respiratory support as determined by the treating clinician (i.e., continuous positive airway pressure (CPAP) or intermittent positive pressure ventilation (IPPV), via facemask or nasal prong/s) in the first 3 min after birth will receive the allocated initial oxygen concentration. If ongoing respiratory support is required, it will be continued using the same allocated oxygen concentration until 3 min of age or until 1 min of respiratory support has been provided, whichever timepoint is later (Fig. 1 a, b). Beyond those time points, respiratory support, including supplemental oxygen, will be administered in accordance with local guidelines, titrated using pulse oximetry.

### Criteria for discontinuing or modifying allocated interventions {11b}

If an infant is severely bradycardic (heart rate < 60 beats per minute) or requires endotracheal intubation, supplemental oxygen will be provided as per local resuscitation guidelines (current international and Australian guidelines recommend increasing the oxygen concentration to 100% in these circumstances).

If an infant commences respiratory support *after* 3 min of age, or if initial respiratory support was ceased and recommenced after 3 min of age, any supplemental oxygen provided will be as per local guidelines, titrated using pulse oximetry as is standard in all participating centers.

### Strategies to improve adherence to interventions {11c}

Strategies to ensure adherence to the treatment pathways include individual site initiation visits, with education provided to all clinicians attending births at those sites, followed by regular education for all new staff at each site. Additional ad hoc training will be provided to sites if requested and at the time of changeover to the alternative treatment pathway, at the commencement of new medical staff, and if there are concerns regarding protocol adherence. To support protocol adherence, color-coded treatment pathway posters and cards attached to oxygen blenders will be placed on every resuscitaire in every delivery room and operating theater in each hospital, as a visual reminder for clinicians. Weekly data reconciliation and monitoring will be undertaken to ensure protocol compliance and complete data collection. Furthermore, the central study team will provide regular communication and support to all participating sites including, but not limited to, regular trial newsletters, site investigator meetings, and site data auditing.

### Relevant concomitant care permitted or prohibited during the trial {11d}

Changes to the set oxygen concentration during the first 3 min after birth, or during the first minute of respiratory support if that commences more than 2 min after birth, are prohibited unless certain criteria are met (see “Criteria for discontinuing or modifying allocated interventions {11b}”). Otherwise, all clinical management is provided at the discretion of the treating team.

### Provisions for posttrial care {30}

Care during and after the infant’s hospital admission, not specified in the trial protocol, will be at the treating clinician’s discretion. In the unlikely event of harm occurring to an infant due to participation in the trial, the central study team will support local site investigators to ensure appropriate treatment and follow-up occurs. Affected families will retain their legal rights to seek compensation.

### Outcomes {12}

#### Primary outcome

The primary outcome is respiratory function measured by the proportion of infants receiving ongoing respiratory support (CPAP or nasal high-flow via facemask, nasal mask, or nasal prong/s; intermittent positive pressure ventilation (IPPV) via facemask, laryngeal mask, or endotracheal tube; or endotracheal intubation and mechanical ventilation) at the time of leaving the delivery room in each treatment pathway (condition).

The primary outcome is electronically entered via a quick response (QR) code link to a secure web-based database by the treating clinical team as soon after stabilization as possible. This will usually occur in the delivery room with the use of the clinician’s smartphone.

#### Secondary outcomes

Some secondary outcomes are collected soon after stabilization as above using the QR code; others are collected at the time of hospital discharge or transfer. Secondary outcomes include the following:

1. Condition at birth as assessed by the 1- and 5-min Apgar scores: Measured by the median Apgar score, reported as a continuous variable at both 1 and 5 min, in each treatment condition
2. Respiratory transition at birth as assessed by the highest level of respiratory support provided in the DR: Measured as an ordinal outcome of infants receiving each level of support ((a) noninvasive respiratory support [CPAP or nasal high flow], (b) IPPV via a noninvasive interface, (c) endotracheal intubation or supraglottic airway insertion with IPPV, or (d) cardiac compressions and/or epinephrine administration). This will be aggregated as the proportion of infants in each category and an odds ratio comparing the treatment conditions.
3. Respiratory transition at birth as assessed by the maximum oxygen concentration provided in the DR: Measured as the mean maximum concentration in each treatment condition
4. Respiratory function post-transition, as assessed by ongoing need for respiratory support beyond the delivery room during the primary hospital admission; measured as age (in days) at final day of (a) endotracheal intubation and ventilation, (b) noninvasive respiratory support, (c) supplemental oxygen, and (d) any respiratory support (endotracheal intubation or noninvasive respiratory support or supplemental oxygen); and measured as the proportion of infants whose age at final day of each respiratory support level was greater than zero in each treatment condition
5. Respiratory distress syndrome as assessed by treatment with exogenous surfactant during the primary hospital admission: Measured by the proportion of treated infants in each treatment condition
6. Respiratory outcome as assessed by the duration of the primary hospital admission: Measured by the mean and median duration of hospitalization in each treatment condition
7. Clinical stability as assessed by the need for interhospital transfer due to escalating care requirements during the primary hospital admission, defined as transfer to a hospital with a higher neonatal capability level [14], and measured by the proportions of transferred infants in each treatment condition.
8. Mortality as assessed by death before primary hospital discharge: Measured by the proportion in each treatment condition

Cost-effectiveness analysis of 30% oxygen and 21% oxygen will be conducted and reported separately from the main trial results. Cost outcomes will include hospital in-patient costs occurring during the birth admission until first discharge home and during the first year of life. Costs will be directly available or estimated using hospital diagnosis-related group data, adjusting for use of oxygen estimated by micro-costing. Effectiveness outcome will include the primary outcome of the trial.

Extended outcomes, available via data linkage, will be analyzed for the subset of infants born in Victoria, Australia, who are consented and enrolled into GenV. These outcomes will be collected external to this trial’s protocol and will be published separately from the AIROPLANE manuscript (Fig. 2).

### Participant timeline {13}

**Fig. 2:**
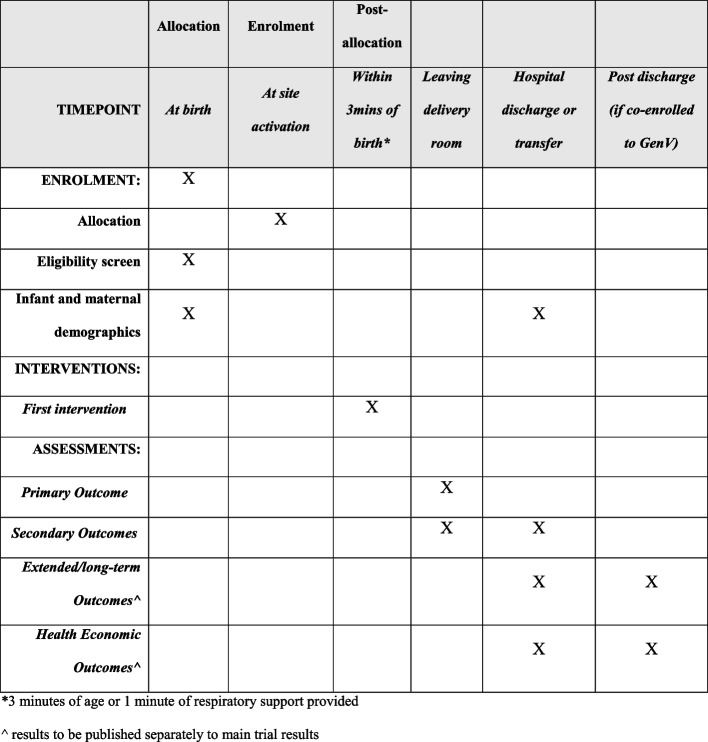
Participant timeline

### Sample size {14}

Based on data from the lead study site (The Royal Women’s Hospital, Melbourne, Australia), 51% of infants born between 32 + 0 and 35 + 6 weeks’ GA who receive respiratory support at birth go on to receive respiratory support beyond the delivery room. An 8% reduction in primary outcome was proposed by consumers and clinicians as a clinically important difference that would drive a change in clinical practice.

To be able to demonstrate an absolute reduction in the primary outcome of 8% (relative reduction 16%), from 51 to 43%, with 80% power and 5% alpha level, with one crossover halfway through each site’s total enrolment period, a minimum of 20 clusters (sites) are required. With an assumed average cluster size of 30 recruits per period, a coefficient of variation of 4, an intracluster correlation coefficient of 0.02, and an exchangeable correlation structure, a minimum of 1200 participants is required from the 20 sites. Sample size calculations were performed using https://clusterrcts.shinyapps.io/rshinyapp/ [15]. If a larger number of clusters and/or a greater sample size are obtained, the power to demonstrate this difference will be increased.

Due to the cluster crossover design, each site will be allocated to provide each study treatment for the same duration of time, from that site’s opening date until the overall trial end date (e.g., 6 months with treatment A and then 6 months with treatment B). Therefore, the study end-point is time-based rather than based on the number of infants enrolled, as long as the total minimum number of infants is achieved from the minimum number of sites. It is therefore likely that the final number of recruited infants will be higher than the minimum required for statistical purposes.

Due to the combination of cluster randomization and waived consent, we anticipate minimal data loss.

### Recruitment {15}

Data from Victoria, Australia, from 2009 to 2017 demonstrated that 35% of infants born at 32 + 0 to 35 + 6 weeks’ gestation received respiratory support at birth. However, the use of respiratory support interventions in the delivery room and beyond is increasing [16]. Unpublished, pre-trial data from the lead study site (2015-20) demonstrated that 45% of infants in the GA range of interest received respiratory support in the delivery room. For study planning, it was therefore estimated that approximately 40% of infants in the GA range of interest would receive respiratory support using study treatments.

In Victoria, Australia, in 2022, 2520 infants were born between 32 + 0 and 35 + 6 weeks’ gestation [2]. Based on 40% receiving respiratory support in the delivery room, we estimated that 1008 infants would be eligible for the AIROPLANE trial per year in Victoria. We aimed to include at least the largest 20 of the 34 Victorian maternity hospitals, which would result in approximately 75% of all Victorian births between 32 + 0 and 35 + 6 being enrolled: equal to 756 infants per year.

A further 3013 infants were born between 32 + 0 and 35 + 6 in New South Wales (NSW), Australia [2], resulting in an estimated 1205 eligible infants (40% of those born in the GA range of interest in NSW) with a plan to invite NSW sites to join the trial if additional centers were required to reach the minimum cluster number needed.

Given the cluster design of the study and the waiver of consent in all but one site, we expect a very high proportion of eligible infants to be enrolled. Eligible infants will be identified by the clinician attending the birth of the infant, followed by automatic enrolment if eligible. To ensure that all eligible infants have their data collected, a process of weekly data reconciliation will occur. This will involve direct communication with every study site to ascertain the number of infants born 32 + 0 to 35 + 6 at that site that week. If the number of births does not match the number of records entered into that site’s study database, any infants with outstanding data entry will be identified and have data entered retrospectively by the local study team.

## Assignment of interventions: allocation

### Sequence generation {16a}

Hospitals will be cluster randomized (all recruits at that site will receive the same study treatment for a set time period), with a single crossover between allocated treatments at each site. Sites will use one treatment pathway for all recruits for the first half of their study recruitment period, before crossing over to complete the second half of their recruitment period using the alternative treatment pathway. Data collected during the first week after the commencement of the second study period after a site has crossed over will not be included in the analysis. This creates a functional “washout” period, allowing for changeover of all study signage and comprehensive staff reeducation. We estimated that the total duration of the AIROPLANE trial would be 32 months from the commencement of recruitment at the first study site. Given that sites will commence recruitment over a period of many months, each site will have a different overall recruitment period (from their site opening date until the overall trial end date). No site will be permitted to join the trial within 12 months of the anticipated trial end date. Sites will crossover at different time points according to when they commenced recruitment relative to the trial end date, to ensure equal time periods recruiting infants via each treatment pathway at each site.

### Concealment mechanism {16b}

This is an unblinded study. Clinicians will set and alter the oxygen concentration being delivered.

### Implementation {16c}

Each hospital will be made aware of their first allocated treatment pathway prior to commencing recruitment, during site education, and by prominent delivery room signage. Clinicians attending births will determine the eligibility of each infant, and the choice of respiratory support is at their discretion.

## Assignment of interventions: blinding

### Who will be blinded {17a}

This is an unblinded study. Clinicians will know the oxygen treatment allocation of their site. The central study team, Trial Steering Committee (TSC), and Data Safety Monitoring Committee (DSMC) will also be unblinded to site allocations. Trial statisticians will be blinded until the statistical analysis plan is finalized.

### Procedure for unblinding if needed {17b}

This is not applicable.

## Data collection and management

### Plans for assessment and collection of outcomes {18a}

The AIROPLANE trial is a clinically embedded, pragmatic study being run in a range of maternity services, including both tertiary and non-tertiary hospitals. Several planned study sites have limited or no research infrastructure or personnel. As such, a minimal amount of essential data will be collected in real time by clinicians caring for eligible infants. Data collection occurs via a scanned QR code linked to a survey and database (REDCap^™^, Vanderbilt University) [17] hosted by the trial sponsor and supported by paper-based backup data forms if required. There are two data collection timepoints: Timepoint 1: soon after birth and immediately post-stabilization and Timepoint 2: at the time of transfer to another hospital or first discharge home from hospital.

Timepoint 1 data is entered by the clinician attending the birth as soon as possible after the delivery, using the REDCap survey accessed via the scanned QR code, and include the following:

- 1. Infant demographics (gestational age, sex, birth weight, multiple birth order if applicable, mode of delivery, delayed cord clamping, Apgar scores at 1 and 5 min of age)
- 2. Maternal demographics (receipt of antenatal corticosteroids in the last 7 days)
- 3. Eligibility as previously described
- 4. Primary outcome as previously described
- 5. Protocol adherence: Oxygen concentration at the commencement of respiratory support, whether the oxygen concentration was changed prior to 3 min of age or 1 min of respiratory support, and, if so, what it was changed to and why
- 6. Resuscitation interventions as previously described
- 7. Receipt of delayed cord clamping, defined as at least 60 s
- 8. The maximum oxygen concentration used at any time in the delivery room
- 9. The infant’s destination upon leaving the delivery room (special care unit/neonatal intensive care unit or postnatal ward)
- 10. Identifiers (date and time of birth and hospital identifier) to enable matching data to Timepoint 2 data entry

Timepoint 2 data is entered by either the clinical team or by research staff, depending on the resources available at each site. Data include the following:

- 1. Secondary outcomes, as previously described, including the last date of any mechanical ventilation, non-invasive respiratory support, supplemental oxygen, and treatment with surfactant
- 2. Infant or maternal demographics not previously entered at Timepoint 1
- 3. Discharge date
- 4. Where applicable, the date, reason, and destination of transfer to another hospital
- 5. If deceased, the date of death and documented cause of death

### Plans to promote participant retention and complete follow-up {18b}

Given the short inhospital follow-up period, with all primary and secondary data collection aimed to be complete prior to hospital discharge, we anticipate 100% ascertainment of the primary outcome and close to 100% follow-up for secondary outcomes. To assist with complete Timepoint 2 data collection, posters will be displayed throughout staff areas in special care nurseries and postnatal wards. Furthermore, each site investigator will receive a weekly email, along with regular contact from the central study team, alerting them to participants with outstanding data.

### Data management {19}

Each site has a named investigator responsible for ensuring data completeness, validation, and reconciliation. Data management training is provided as part of each site’s initiation visit. Data reconciliation will occur weekly via contact with the investigator or delegate at each site to ensure complete and valid data entry for all eligible infants. Duplicate and incorrect entries will be identified and addressed, and any protocol deviations will be logged. Central monitoring will occur regularly to perform targeted source data verification and ensure study compliance. All data will be stored on a secure password-protected REDCap database managed by MCRI. All data will be securely retained for 25 years and destroyed in a secure manner thereafter, as per NHMRC guidelines [18].

### Confidentiality {27}

Participant confidentiality will be maintained by the site investigators, research staff, and sponsor under data protection and privacy laws. Patient identifiers will be anonymized prior to data analysis. No data will be released to any unauthorized third party without prior written approval from the sponsoring institution. All data will be anonymized in any publications related to the trial.

### Plans for collection, laboratory evaluation and storage of biological specimens for genetic or molecular analysis in this trial/future use {33}

This is not applicable.

## Statistical methods

### Statistical methods for primary and secondary outcomes {20a}

The primary outcome, whether the infant is receiving respiratory support when leaving the delivery room, will be analyzed as a binary outcome. The incidence of this outcome will be summarized as the number and percentage in each treatment arm.

The treatments will be compared using a risk difference and 95% confidence interval (CI) using individual participant level data. A cross-sectional sample in each treatment period will be modelled with a mixed-effects generalized linear model (GLM) with an identity link function and a binomial distribution using an exchangeable correlation structure to model the correlation within each cluster, adjusted for treatment period due to the crossover nature. The treatment group effect and period effect will be fixed, and the cluster effect will be random. The primary analysis will be by intention to treat and will comprise all eligible infants from every study site, regardless of adherence to the trial protocol. Exclusions will consist only of participants whose parents requested to have their child’s data removed, participants enrolled during the “washout period” (defined as the first week after treatment crossover at each site), and participants included in error (ineligibility criteria identified post inclusion).

Dichotomous secondary outcomes will be analyzed in the same manner. Continuous secondary outcomes will be analyzed in a similar manner with models using a Gaussian distribution rather than a binomial distribution. Ordinal secondary outcomes will be analyzed using ordinal logistic regression.

A statistical analysis plan will be published separately to this protocol prior to the analysis of the results. There are no interim analyses planned.

### Interim analyses {21b}

No interim analyses are planned. An independent Data Safety and Monitoring Committee (DSMC) reviewed mortality in the trial after 600 participants had been recruited (50% of the minimum planned sample size). A recommendation to terminate the trial early could be made if there was a higher-than-expected mortality rate, inadequate trial resources, a diminished significance of the study question, or other evidence raising ethical concerns. The DMSC recommended continuation of the trial, unchanged, after this review.

### Methods for additional analyses (e.g., subgroup analyses) {20b}

There are no planned subgroup analyses.

### Analysis population and statistical methods to handle missing data {20c}

The primary analysis will be based on the intention-to-treat population. Missing data are not anticipated, as explained in Sections 18 and 19, but if more than 10% of the outcomes are missing, multiple imputation will be used to handle missing data.

### Plans to give access to the full protocol, participant level-data and statistical code {29, 31c}

Information regarding the trial is publicly available at the Australian New Zealand Clinical Trials Registry (www.anzctr.org.au), as well as on the AIROPLANE trial website (www.airoplanetrial.org.au). Participant-level data will not be made available under the conditions of the consent waiver. As such, there is no sharing of participant data possible.

## Oversight and monitoring

**Table:** 

**Trial Steering Committee**	
A/Prof. Louise Owen	Royal Women’s Hospital, Melbourne
(Principal Investigator)	Clinical Sciences, Murdoch Children’s Research Institute, Melbourne
	Department of Obstetrics, Gynaecology and Newborn Health, The University of Melbourne, Melbourne
Dr. Stacey Peart	Royal Women’s Hospital, Melbourne
(Chief Investigator)	Clinical Sciences, Murdoch Children’s Research Institute, Melbourne
	Department of Obstetrics, Gynaecology and Newborn Health, The University of Melbourne, Melbourne
A/Prof. Brett Manley	Mercy Hospital for Women, Melbourne
(Chief Investigator)	Clinical Sciences, Murdoch Children’s Research Institute, Melbourne
	Department of Obstetrics, Gynaecology and Newborn Health, The University of Melbourne, Melbourne
Prof. Peter Davis	Royal Women’s Hospital, Melbourne
(Chief Investigator)	Clinical Sciences, Murdoch Children’s Research Institute, Melbourne
	Department of Obstetrics, Gynaecology and Newborn Health, The University of Melbourne, Melbourne
Prof. Jeanie Cheong	Royal Women’s Hospital, Melbourne
(Chief Investigator)	Clinical Sciences, Murdoch Children’s Research Institute, Melbourne
	Department of Obstetrics, Gynaecology and Newborn Health, The University of Melbourne, Melbourne
Prof. Ju-Lee Oei	National Health and Research Medical Council Clinical
(Chief Investigator)	Trials Centre, The University of Sydney, New South Wales, Australia
	Department of Newborn Care, The Royal Hospital for Women, Randwick, New South Wales, Australia
	Discipline of Paediatrics and Child health, School of Clinical Medicine, University of New South Wales, New South Wales, Australia
Dr. Jubal John	NeoPaeds Melbourne, Melbourne
(Site Investigator)	Joan Kirner Women’s and Children’s Hospital,
Dr. Niranjan Abraham	Melbourne
Dr. David Tickell	Ballarat Base Hospital, Melbourne
(Site Investigator)	Clinical epidemiology and Biostatistics Unit, Murdoch
Dr. Anneke Grobler	Children’s Research Institute, Melbourne
	Department of Paediatrics, The University of Melbourne, Melbourne
Prof. Andrew Davidson	Murdoch Children’s Research Institute, Melbourne
(Trial Sponsor)	
A/Prof. Andrew Gill	King Edward Memorial Hospital, Perth
(Independent Clinical Expert)	Department of Paediatrics, University of Western Australia, Perth
Dr. Trisha Prentice	Royal Children’s Hospital, Melbourne
(Independent Clinical Expert	Clinical Sciences, Murdoch Children’s Research
and Ethics Advisor	Institute, Melbourne
Ms. Catherine Beesley	Consumer Advisory Group, Centre of Research
(Consumer Representative	Excellence in Newborn Medicine, Melbourne
Dr. Li Huang	Melbourne School of Population and Global Health
(Health Economist)	The University of Melbourne, Melbourne
Ms. Renae Allen	Melbourne Children’s Trial Centre, Murdoch Children’s
(Trial Coordinator)	Research Institute, Melbourne
**Data Safety and Monitoring Committee**	
Prof. Adam Buckmaster	Chair & Independent Expert, Women, Children and Families, Central Coast Local Health District, NSW
Prof. Andrew McPhee	Independent Expert, Women’s and Children’s Hospital, Adelaide
Dr. Anurika De Silva	Independent Statistician, Melbourne School of Population and Global Health, The University of Melbourne, Melbourne

### Composition of the coordinating center and trial steering committee {5d}

The central study team is based at The Royal Women’s Hospital (RWH) and the MCRI, Melbourne, Australia, and meets fortnightly. The TSC, consisting of the chief investigators, site investigators or delegates, consumer representative, independent clinical expert, ethics advisor, and trial statistician, meets three times per year and provides oversight and leadership of the trial.

### Composition of the data monitoring committee and its role and reporting structure {21a}

The independent DSMC consists of the DSMC Chair (an independent expert pediatrician/triallist), a second independent expert neonatologist/triallist, and an independent statistician. The DSMC is responsible for the stewardship of the trial. This includes the review of site recruitment, accrual, retention, and withdrawal, as well as protocol adherence, data management, and quality control. The DSMC will be responsible for ensuring the safety and interests of trial participants, which will be exercised by providing recommendations regarding the continuation, modification, or termination of the trial on safety grounds. The DSMC met at study commencement and conducted a safety review after 600 recruits. The DSMC recommended the continuation of the trial, unchanged, after this review.

### Adverse event reporting and harms {22}

This study has been deemed to be very low risk. No adverse events or serious adverse events have been defined. As previously outlined, the DSMC reviewed the incidence of death after 600 recruits.

### Frequency and plans for auditing trial conduct {23}

Trial monitoring will occur regularly and continuously by the coordinating study team, to ensure protocol adherence and reporting of protocol deviations by site, and to ensure data completeness and validity for all eligible infants. Additionally, central auditing (monitoring) against source data will be performed on a sample of participants at each site approximately 12 weeks after the first recruit is enrolled and annually thereafter.

The first monitoring visit will verify data entry against source data for the first 10 participants enrolled at each site or the total number of participants enrolled if less than 10. Annual reviews thereafter will review source data from a random selection of participants totalling 10% of participants enrolled per annum or a minimum of 10 recruits per annum. Sites with a high proportion of protocol deviations and/or retrospective data entries will have a review of 20% of recruits, randomly selected, with a minimum of 10 participants’ reviews; if < 10 participants are recruited, all recruits will be reviewed.

### Plans for communicating important protocol amendments to relevant parties (e.g., trial participants and ethical committees) {25}

Any protocol amendments will be submitted for approval by The Royal Children’s Hospital Human Research Ethics Committee, Melbourne, and the Mercy Health Human Research Ethics Committee, Melbourne. Any approved changes will be communicated to all site principal investigators to obtain site-specific governance approval.

#### Dissemination plans {31a}

The trial steering committee will distribute trial information to participating sites via distribution lists and email correspondence. The results of this study will be presented at national and international conferences and published in a peer-reviewed journal. Results will also be communicated to consumers and stakeholders via presentations and the study website (airoplanetrial.org.au).

## Discussion

The optimal initial oxygen concentration with which to begin respiratory support in moderate-late preterm infants in the minutes after birth remains unknown. The AIROPLANE trial will address this gap in the evidence. This is a pragmatic study, embedded in clinical practice and recruiting in both experienced research hospitals and sites that do not have access to research infrastructure or personnel. To ensure feasibility, this cluster-randomized study, operating under a waiver of consent, is employing a novel data collection strategy using a scanned QR code to allow clinicians to enter minimal data in real time with minimal interruption to clinical work. This trial is powered to detect an important difference in rates of ongoing need for respiratory support upon leaving the delivery room. The findings of this study may impact national and international resuscitation guidelines to improve the care of moderate-late preterm infants.

### Trial status

The trial is currently operating under protocol version 6.0, dated 13th February 2024. Recruitment commenced on 14th December 2022 at The Royal Women’s Hospital, Melbourne, Australia, and is currently recruiting in a total of 26 sites across Victoria and New South Wales, Australia. At the submission of this protocol for publication in March 2025, recruitment had reached 1357 participants. The trial is planned to complete in September 2025. The DSMC safety review was conducted in July 2024; no concerns were raised, nor were amendments required. Final study sites were opened in late 2024, after which the planned submission of this protocol occurred in February 2025.

## Data Availability

Anonymized data collected for analysis will be available 6 months after publication of the primary outcome. An application to obtain the data may be made by contacting MCTC@mcri.edu.au. No data will be released to a third-party representative without written approval by the trial sponsor.

## Abbreviations

CI: Confidence interval
CPAP: Continuous positive airway pressure
CRE: Centre of Research Excellence
DSMC: Data Safety Monitoring Committee
ERC: European Resuscitation Council
GA: Gestational age
GenV: Generation Victoria
GLM: Generalized linear model
HREC: Human Research Ethics Committee
ILCOR: International Liaison Committee on Resuscitation
IPPV: Intermittent positive pressure ventilation
MCRI: Murdoch Children’s Research Institute
NHMRC: National Health and Medical Research Council (NHMRC
NSW: New South Wales
QR: Quick response
RWH: Royal Women’s Hospital
TSC: Trial Steering Committee
VIC: Victoria
